# Prospective, Clinical Pilot Study with Eleven 4-Mm Extra-Short Implants Splinted to Longer Implants for Posterior Maxilla Rehabilitation

**DOI:** 10.3390/jcm9020357

**Published:** 2020-01-28

**Authors:** Daniel Torassa, Pablo Naldini, José Luis Calvo-Guirado, Enrique Fernández-Bodereau

**Affiliations:** 1 Specialist Program in Removable and Fixed Prostheses and Implantology, Faculty of Dentistry, National Universidad Nacional de Córdoba (UNC), Córdoba 5000, Argentina; pnaldini@hotmail.com (P.N.); fernandezenrique5@gmail.com (E.F.-B.); 2 Department of Oral and Implant Surgery, Faculty of Health Sciences, Universidad Católica de Murcia (UCAM), 30107 Murcia, Spain; jlcalvo@ucam.edu

**Keywords:** extra short implant, short implant, splinted, posterior maxillae, treatment protocol, osseointegration enhancement

## Abstract

In many clinical situations, rehabilitation with implants in the posterior maxillary region is complicated by limited bone availability. In this context, the use of 4 mm long implants (known as extra-short implants) may be used thanks to the concept of osseointegration enhancement. It has been demonstrated that short implants offer an alternative to the regeneration procedures involved in placing longer implants in areas where bone height is compromised. This prospective pilot study tested a treatment protocol in which 11 extra-short (4 mm) implants were splinted to 11 mesially placed longer (8 mm) implants in the posterior maxillary regions of partially edentulous patients, without using supplementary bone regeneration procedures. Eleven patients were included in this single cohort study. The clinical performance of the extra-short implants was assessed during a two-year follow-up period, obtaining a 100% survival rate and mean bone loss of 0.3 mm. Implant stability measured by resonance frequency analysis (RFA) at the time of placement was 54.9 ± 4.9, increasing to 77.0 ± 2.6 at 24 months. The study demonstrated the gradual consolidation of osseointegration in bone of less-than-ideal quality in the posterior maxillary region. The results obtained show that a partially edentulous maxilla with reduced bone height may be rehabilitated by using an extra-short implant splinted to a mesial implant of 8mm length or longer. Despite the small sample size, this pilot study observed that extra-short implants achieved adequate bone stability and clinical performance after a 24-month follow-up.

## 1. Introduction

Osseointegration enhancement aims to exploit the possibilities offered by advances in implant system design, surface treatments, and the strength of materials so they make optimal use of the biological resources available in the individual patient, especially the bone terrain, which is often limited in height or in buccolingual volume. This involves the use of implants of reduced length (short or extra-short implants) or reduced diameter (small- or narrow-diameter implants) providing the availability of bone is sufficient to permit the required prosthetic rehabilitation. The use of 4 mm long (or extra-short) implants [1] can simplify treatment in a range of clinical situations, while reducing morbidity and economic cost. Moreover, extra short implants help meet the aims of contemporary implant dentistry, namely, to minimize the invasiveness of procedures, and to reduce treatment time and economic cost while ensuring successful and predictable outcomes. In the last ten years, the use of short implants has increased significantly, especially in partially edentulous maxillae, but information regarding extra-short implants (<7 mm) remains limited [2]. In the posterior maxilla, the alveolar ridge may present limited bone availability for anatomical reasons or as a result of bone remodeling, conditions that can be overcome through the use of short implants. This possibility is of particular interest in the posterior maxillary region, due to its proximity to the maxillary sinus, where implant placement often necessitates some bone regeneration procedure, leading to longer treatment time, possible complications, morbidity, and increased economic cost. The accepted procedures for creating the necessary conditions for the placement of longer implants in the posterior maxillary region adjacent to the maxillary sinus include sinus floor lift by the transcrestal technique (when residual bone should be of sufficient width and present a height of at least 5 mm), and the lateral window technique, which makes it possible to fill or graft a range of biomaterials to increase the available height for implant placement [3,4]. But maxillary sinus floor lift is an invasive procedure with associated morbidity, and involves a considerable prolongation of treatment time and increased economic cost [5,6,7,8,9]. In some clinical situations, it is necessary to increase the caudal vertical bone height, i.e., below the floor of the maxillary sinus. This is achieved by means of complex surgical procedures for vertical regeneration [10,11].

Many studies have shown that the rehabilitation of partially edentulous patients using short implants of up to 6 mm can be as successful as rehabilitation using longer implants in both maxillae [12,13,14,15,16]. A 2017 meta-analysis reviewed seven studies comparing short implants with longer implants placed using maxillary sinus floor lift; the short implants showed a significantly lower rate of complications, with a success rate that was comparable to the longer implants [5]. In the posterior maxilla, at least 6 mm of residual bone height below the floor of the maxillary sinus is usually necessary but with 4-mm extra-short implants, the residual bone needed can be even less. Survival values for implants that are 4.0 mm to 5.4 mm in length show adequate results in both maxilla and mandible [17]. Nevertheless, these results, although promising, are insufficient in terms of the number of implants evaluated and the duration of follow-up periods. When short implants are used, doubts arise regarding biomechanical aspects, particularly their capacity to withstand masticatory forces. These doubts are even greater when extra-short implants are used. Studies using FEA (finite element analysis) of short implants have observed adequate biomechanical capacity in terms of the resistance to and distribution of forces compared with longer implants. In these studies comparing short implants with standard long implants, the application of forces oblique to the implants resulted in a similar stress concentration at the cervical region, and no changes in bone stress were observed due to an unfavorable crown-to-implant ratio; however, changes were observed in prosthetic component stress derived from the unfavorable crown-to-implant ratio [18,19,20]. Systematic reviews regarding the crown-to-implant ratio in single-tooth, non-splinted implants have not found any differences in complication rates between implants of up to 6 mm and longer implants [21]. 

As yet, clinical trials have not produced any data regarding the survival and success rates of rehabilitation in the posterior maxillary region using 4-mm short implants splinted to implants of greater length. Investigating this rehabilitation method with splinted components, some studies of cantilever techniques using finite element analysis (FEA) have found no differences in stress concentrations between 8 mm and 4 mm implants, a finding that which suggests the clinical potential of the latter [22].

Treatment protocols using 4-mm implants in the mandible have already been established in previous research [23,24,25]. But to date no results have been published for protocols using extra-short implants splinted to implants of greater length in the maxilla. The purpose of this clinical study was to evaluate the use of extra-short (4 mm) implants splinted to 8 mm implants placed in the posterior maxilla as a means of overcoming limited bone availability, and determine how splinting to longer implants might influence outcomes for the short implants in the short- and medium term. In particular, the objectives were, firstly, to evaluate the survival rate of extra-short implants (4 mm), to analyze crestal bone stability, and to evaluate implant stability by RFA; and secondly, to establish the use of extra short implants as an alternative to accepted options such as sinus floor lift or ridge split technique.

## 2. Experimental Section

The local Institutional Health-Care Research Ethics Committee (CIEIS) approved the study protocol (Reg. No. 6 I), which was conducted at the School of Dentistry of the National University of Cordoba (Argentina). Before the study commenced, informed consent was provided and signed by all patients.

### 2.1. Patients

A total of 22 implants were placed consecutively at healed sites in 11 patients, nine females and two males of ages ranging from 42 to 69 years (mean age 57 ± 8.4 years). All patients were restored with 11 8-mm length implants in mesial position, splinted to 11 extra-short implants of 4-mm length in distal position located in the posterior maxilla. Treatment evolution was monitored regularly over a two-year follow-up period (Table 1).

The design of the project was as follows: 

Follow-up visits were scheduled to take place two months after implant placement when provisionalization was performed, at six months when final restorations were placed, and at 12 and 24 months.

Inclusion criteria were as follows: (1) patients aged between 20 and 65 years and in good health; (2) patients willing to participate in the study for the full duration demonstrated by providing their informed consent; (3) patients presenting partially edentulous posterior maxillae in the molar/premolar region with sufficient bone height and volume for insertion of 4 mm long, 4.1 mm diameter implants; and (4) soft tissues free of mucosal lesions, dental caries, and periodontitis at the time of implant insertion. 

Exclusion criteria were as follows: (1) presence of uncontrolled diabetes mellitus; (2) alcohol or drug abuse; (3) systemic disorders that could jeopardize implant placement; (4) insufficient bone for placement of a 4 mm long implant; (5) previous bone graft procedures in the area under investigation; (6) pregnancy; (7) previous radiation therapy to the head or neck; and (8) chemotherapy within five years prior to surgery. Neither cigarette smoking nor bruxism were considered contraindications for treatment, although these factors were recorded. Primary stability was assessed by resonance frequency analysis (RFA) using an Osstell device at the time of implant placement and at two months, 6, 12, and 24 months after implant placement. 

Bone remodeling was determined using standardized periapical radiographs at the time of implant placement, and at two months, 6, 12, and 24 months thereafter.

### 2.2. Surgical Procedure

Preoperative antibiotic treatment consisted of 875 mg amoxicillin (Amixen Duo, Laboratorios Bernabó, CABA, Argentina) in two doses every 12 h for seven days; in case of allergy to penicillin, 600 mg of clindamycin was prescribed.

Painkillers and an analgesic loxoprofen (Tabe, Laboratorios Bernabó, CABA, Argentina) were prescribed every 12 h for 48–72 h as required.

The protocol requires adequate residual ridge resorption conditions to allow implant placement, with residual bone beneath the maxillary sinus no less than 6 mm wide and 4 mm high. The mean height measured during preliminary diagnostic examinations was 4.5 mm from the maxillary sinus floor to the ridge crest.

**Figure 1:**
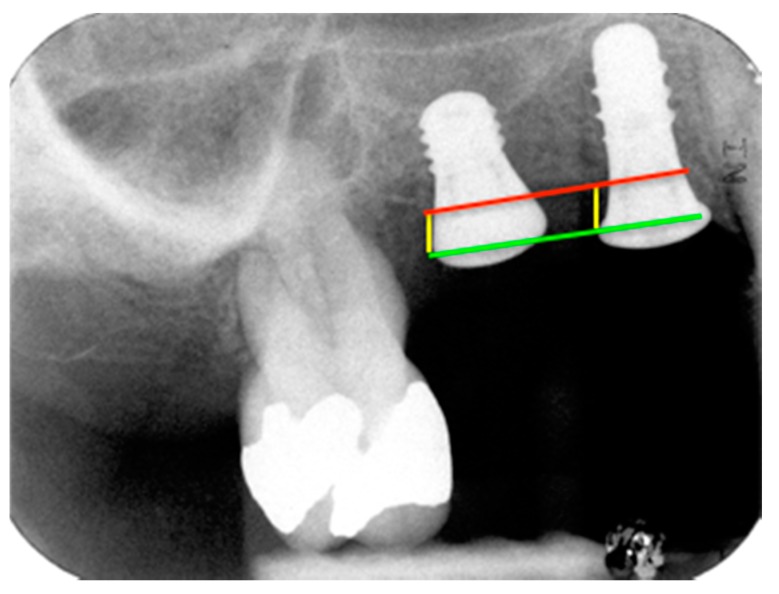
References for MBL and CBL measurements: CBL, red; implant shoulder (IS), green; implant shoulder to first point of bone-to-implant contact (IS-BIC), yellow.

In all 11 cases, Straumann implants of 3.3 mm or 4.1 mm in diameter by 8 mm length were placed in mesial position (Standard Plus implant, RN, Roxolid^®^, SLActive^®^, Institut Straumann AG, Basel, Switzerland). Extra-short implants of 4.1 mm in diameter and 4 mm length were placed in distal positions in the edentulous area (Standard Plus implant, 4.1 mm RN, Roxolid^®^, SLActive^®^ 4 mm. Institut Straumann AG, Basel, Switzerland) using the drilling sequence recommended by the manufacturer. References for measuring marginal bone level (MBL) and crestal bone loss (CBL) are illustrated in Figure 1.

### 2.3. Calibration

Radiographic images were spatially calibrated with Image Pro-Plus v.4.5 software (Media Cybernetics Inc., Rockville, MD, USA) to minimize error or bias in insertion level measurements, which could have been produced by geometric distortion due to non-parallelism of the radiographic plate. Spatial calibration (indirect method) of the digital images was carried out by establishing the relationship between numbers of pixels and the known length of the implant of smaller size (5.8 mm long including the 1.8 mm polished neck).

After measuring the alveolar ridge’s mucosa thickness, a crestal incision was made to raise a full-thickness flap without compensatory incisions, of sufficient extent to allow implant placement in the edentulous space. 

**Figure 2:**
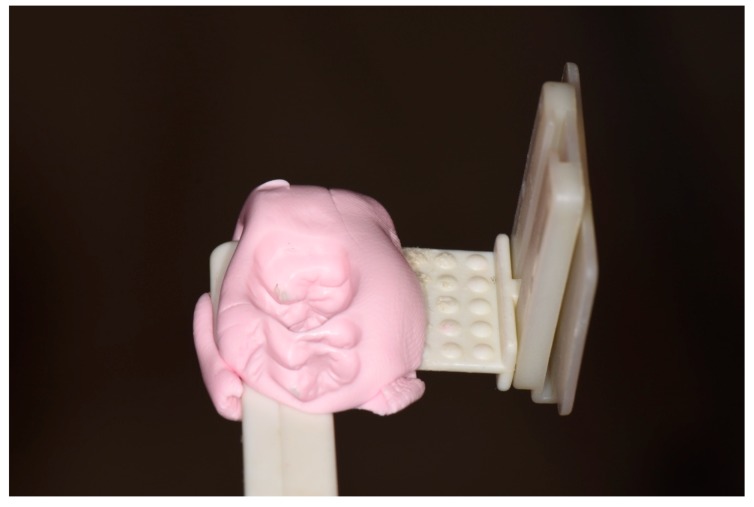
Custom bite blocks for periapical radiographs.

Standardized periapical radiographs were taken using a single device (custom bite blocks and the paralleling technique) for each patient (Figure 2). 

Distal and mesial crestal bone levels were measured by determining the distance from the reference point on the implant (the implant shoulder) to the first point of bone-to-implant contact (Figure 1). It should be noted that the tissue level implants used in the study have a 1.8 mm unroughened titanium surface, so the length measured includes this distance. The differences between MBL values at baseline and each evaluation stage were calculated, recording the changes to MBL at 12 and 24 months after implant placement. The radiographs were scanned using an HP G-3110 photo scanner (Palo Alto, California, CA, USA) with a resolution of 1200 dpi. The images captured were then processed and measured with Image-Pro Plus image processing software v.4.52 (Media Cybernetics Inc., Rockville, MD, USA). 

Resonance frequency analysis (RFA) was used to assess implant stability using an Osstell device (Osstell AB, Gothenburg, Sweden) [26,27].

**Figure 3:**
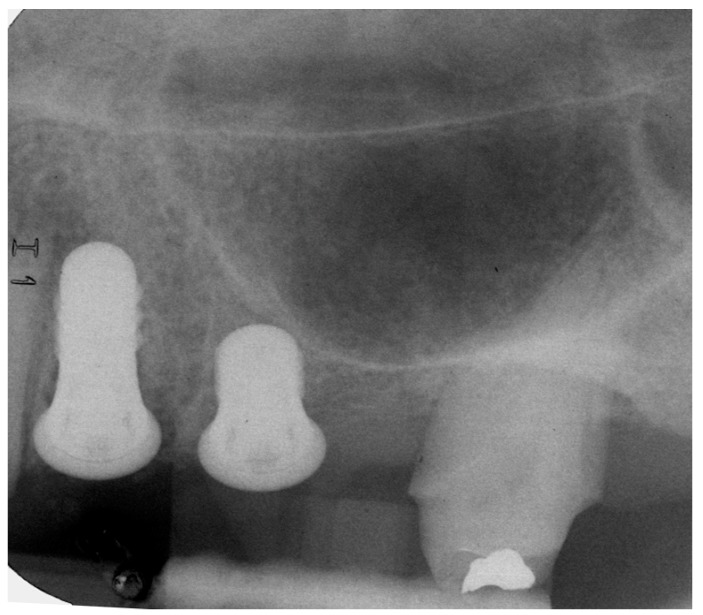
Radiograph of extra short and long implants at implant placement.

**Figure 4:**
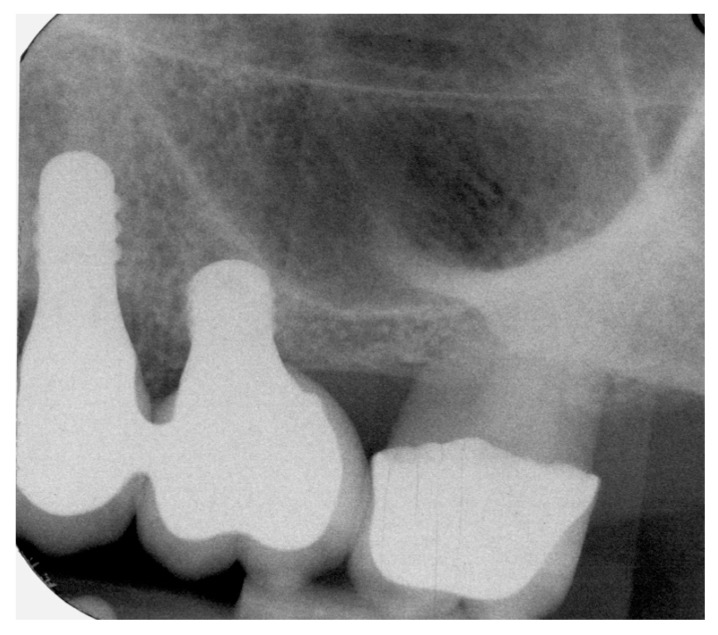
Radiograph of extra short and long implants at 12-month follow-up.

**Figure 5:**
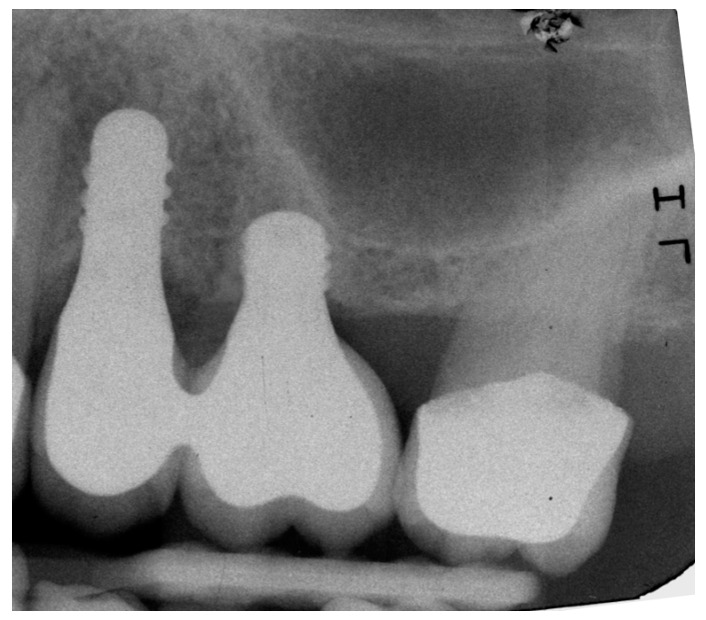
Radiograph of extra short and long implants at 24-month follow-up.

The measurements were taken immediately after implant placement in the vestibular and lingual positions perpendicular to SmartPeg rods (SmartPeg; Osstell AB, Gothenburg, Sweden), placed on the implants. The 22 implants’ ISQ (Implant Stability Quotient) was measured at five stages: at baseline, and at 2, 6, 12, and 24 months after implant placement [28,29]. Sutures were removed two weeks after implant placement. Two months later, provisional restorations were placed on temporary abutments, and RFA and radiographic examination were performed as described above. At six months the definitive restorations were placed, all restorations were veneered crowns cemented and screwed onto variobase abutments. Patients returned for follow-up examinations at 12 months and 24 months after implant placement (Figure 3, Figure 4 and Figure 5).

**Figure 6:**
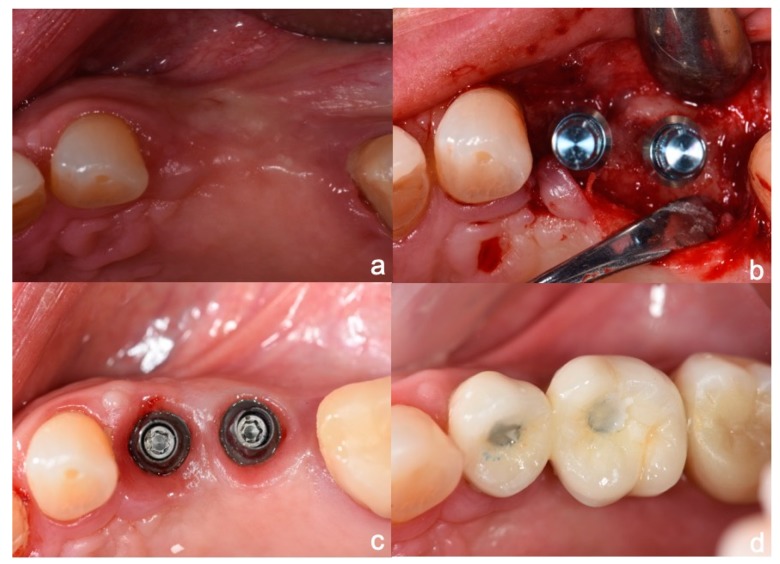
(**a**) Clinical spam of missing teeth; (**b**) long and extra-short implants in place; (**c**) variobase abutments screwed to the implants; (**d**) ceramic crowns in place.

**Figure 7:**
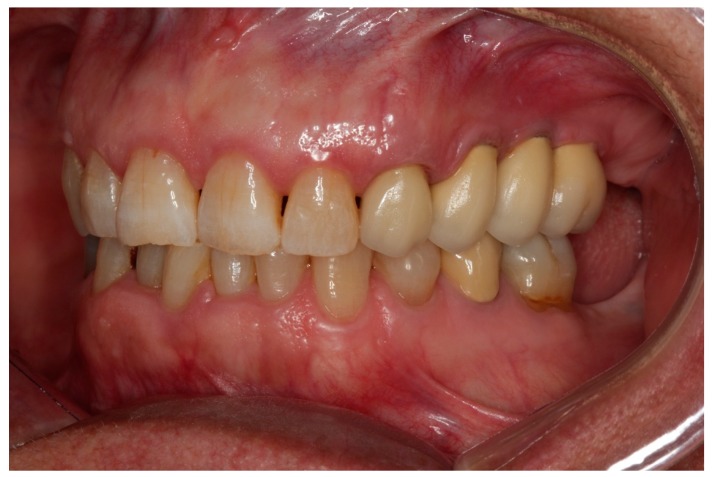
Lateral view of implant crowns.

As all the clinical cases presented partial edentulism in premolar and first molar areas, soft tissue harmony was a matter of great importance (Figure 6). However, this was not the subject of the present pilot study, so data regarding soft tissue harmony was not recorded. The 11 cases rehabilitated suffered no major resorption of the ridge’s vertical plane but pneumatization of the floor sinus was observed. In the horizontal plane, mostly in the molar area, some overcontouring of the crowns’ vestibular plane resulted in a complaint made by one patient of food retention, which was solved by reducing the vestibular overcontouring (Figure 7).

If any complications or complaints regarding the devices or procedures arose, they were recorded at each follow-up visit or restoration stage. These were listed as adverse events in the patient’s medical records. 

Patients’ degree of satisfaction was evaluated by means of four quick and easily answered questions, as follows:

l. Did you feel any type of pain during treatment?

2. Do you have any problems when speaking or laughing related to the implant crowns or prostheses?

3. Can you eat comfortably with the new prostheses?

4. Would you like to repeat the implant treatment? 

For the four questions the following scores was awarded: one for affirmative answers, two for ambiguous answers and three for negative responses. The following range was established to quantify overall satisfaction: 1–4 = dissatisfaction; 5–9 = partial/regular satisfaction; 10–15 = complete satisfaction. To analyze patient satisfaction, the results were expressed as percentages and subsequently different variables were related to patient satisfaction using the chi-squared test (degree of significance: *p* < 0.05). 

Ninety percent of patients did not report having any problems when speaking and eating. No patient (93.5%) had problems when laughing. Most of the patients (95.1%) included in the study could eat comfortably with their new prosthetic rehabilitation. Two patients reported food debris penetration between crown and teeth. Regarding the degree of overall satisfaction, 95% of patients were satisfied with the treatment they had received and its outcomes.

Implant survival was defined as presence of the implant in function without pain, absence of mobility or bone remodeling evaluated from conventional radiographs. Other clinical parameters assessed were BOP, presence/absence of inflammation or suppuration under visual examination.

## 3. Statistical Analysis

Data were analyzed with SPSS software v.19 (IBM SPSS Statistics, Chicago, IL, USA). Descriptive statistics were calculated for bone stability measurements and resonance frequency analysis: central tendency (mean) and dispersion (standard deviation). Subsequently, implant groups (short and long) were compared to identify differences between the bone levels recorded during the examinations, using non-parametric tests (Mann–Whitney U test). In addition, the distributions of values at the five stages of the study (0, 2, 6, 12, and 24 months) were compared using the Kruskal–Wallis test. 

### Statistical Power

Statistical power was calculated considering both the level of significance established in the study (α = 0.05), the standard deviation values of both parameters observed at each stage, and the number of controlled cases (which decreased as a function of time) (Table 2). For implant stability analysis (ISQ), the study considered differences between groups equal to or greater than 5 ISQ (dISQ = 5), while for bone remodeling analysis it considered differences between implants greater than 0.5 mm (dRem.v. = 0.5). In each case, statistical power was obtained from the values of Z (1-β) using the following formula:

that for α = 0.05 results Z_(1-α)_ = 1.96 (1)

## 4. Results

No implant failures occurred within the 24 months of follow-up and so the implant survival rate for both 8-mm and 4-mm implants was 100%.

At the time of implant placement (baseline), mean ISQ was 64.4 for the 8-mm implants, while 4mm implants obtained a lower mean value (ISQ = 54.9). This value increased throughout the follow-up period, reaching the level of the ISQ values for 8-mm implants by 24 months (Table 3).

**Figure 8:**
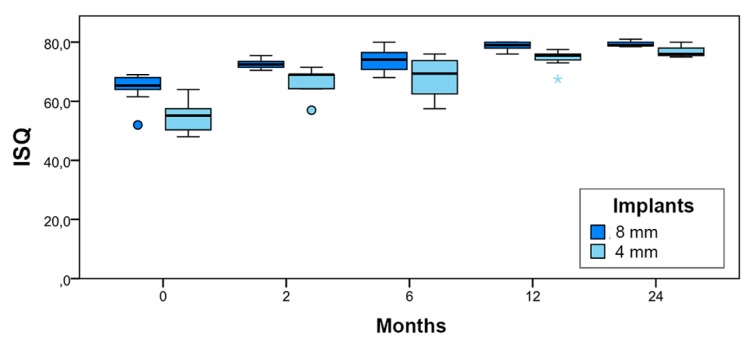
Schematic distribution of ISQ values by evaluation time and implant type.

Differences in ISQ values were not statistically significant at the 6-month and 24-month evaluations (*p* > 0.05), but were significant at other evaluation times (baseline, two months, and 12 months) (*p* < 0.05). In the box plot shown in Figure 8, distributions of ISQ values for both groups (long and short) are represented schematically at each evaluation time.

### 4.1. Marginal Bone Level (MBL)

Marginal bone level was measured on both sides of each implant at each evaluation time. 

As described in Materials and Methods, measurements were taken from the implant shoulder to the first point of bone-to-implant contact, so the values were all positive numbers.

To calculate changes in MBL, the 1.8 mm of polished neck was subtracted from the total distance.

Table 4 shows MBL values corresponding to each implant group and evaluation time. Although at baseline the MBL value for 8-mm implants was higher than that of 4-mm implants; at later stages (12 and 24 months) 4-mm implants showed higher MBL values. However, at all evaluation times differences between implant groups were not significant (*p* > 0.05).

### 4.2. Bone tissue remodeling (MBL change)

Changes in MBL indicated the extent of bone remodeling in comparison with baseline. Table 5 shows changes in MBL in both implant groups at each evaluation time. The differences between implant groups were not statistically significant at any stage (*p* > 0.05).

**Figure 9:**
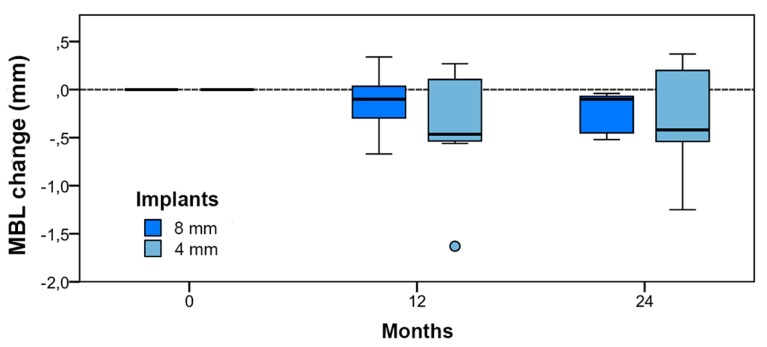
Bone tissue remodeling value distribution by stage and implant type.

Taken together, the differences between evaluation times were significant (Kruskal–Wallis: *p* < 0.05), but only in comparison with baseline, and not between 12 and 24 months (paired comparison; two-tailed tests: a) 0–12 months *p* < 0.05; b) 0–24 months *p* < 0.05; and c) 12–24 months *p* > 0.05). In the box plot shown in Figure 9, the distributions of remodeling values for the two implant groups (long and short) are represented schematically at each evaluation time.

## 5. Discussion

Rehabilitation in the maxillary posterior region, where atrophy is a three-dimensional process, may involve ridge height and width augmentation beneath the maxillary sinus whenever ridge dimensions are insufficient to allow implant placement. The results obtained in the present trial suggest that clinical situations of partially edentulous spaces in the posterior maxilla requiring the replacement of two teeth may be treated successfully by splinting an 8-mm long implant to a 4-mm extra-short implant. In the present work, no implant failures occurred during the two-year follow-up period, a finding that vouches for the efficacy of this treatment protocol. 

For the extra-short implants investigated, the mean RFA value of 54.2 ISQ measured at the time of implant placement (baseline) demonstrates the difficulty of obtaining initial stability in maxillary posterior regions, as this was considerably lower than mean values recorded in studies of the mandible where bone characteristics favor initial stability [6,7,8,9,25,26,30]. Primary implant stability was obtained following a conventional drilling protocol, which may be further enhanced by underpreparing the osteotomy and osteotomes [31,32]. Moreover, as argued by Qian et al., RFA values should not be considered as determinants of the loading time point [33]. In any case, the present study found that the low mean RFA value for short implants recorded at the time of placement evolved towards stability similar to that of the long implants six months after placement, demonstrating their capacity for osseointegration; data recorded from two months onwards (when provisionalization began) showed an increase in secondary stability. As stated in the Results section, no differences were detected at six and 24 months between the two implants, although at the six-month evaluation time, non-significance may have been due to the high dispersion of stability values. If the treatment protocol described is to be applied, residual ridge resorption conditions must be sufficiently favorable to allow implant placement, with residual bone beneath the maxillary sinus no less than 6 mm wide and 4 mm high. The choice of treatment will be determined by this diagnostic assessment. 

In many cases, treatment will require preparation of the bone area in all three dimensions to compensate for the contour changes the ridge has undergone. With regard to residual height, the literature states that for procedures in which one of the elevation techniques outlined above was performed prior to implant placement, the mean height of sub-antral bone was 3.8 mm, while in cases in which sinus lifting was performed using the lateral window technique with simultaneous implant placement, mean height was 4.4 mm and combine with distractors [34]. In the present study, mean height from the maxillary sinus floor to the ridge crest measured in preliminary diagnostic examinations was 4.5 mm, a sufficient amount to permit the placement of 4-mm implants in the distal portion of the edentulous area without any need to carry out regeneration procedures to augment ridge height. In lifting procedures using the lateral window technique with simultaneous implant placement, implant loading times vary from 3 to 4 months after the start of treatment, but with extra-short implants these times can be reduced to 2 months after implant placement. Complications reported for the lateral lifting technique are mainly biological, for example, sinus membrane perforation, and they are nearly three times more frequent in comparison with short implants [35]. Operative time, patient-reported outcomes, morbidity, and economic costs have been found to be more favorable for short implants than lateral lifting [36]. 

With regard to transcrestal sinus floor elevation using osteotomes, which could be an alternative to the protocol proposed here, no randomized comparative studies have been published that investigate extra-short implants. For transcrestal techniques, a residual ridge height of at least 5 mm is necessary, although Nedir et al. [37], placed 8 mm implants in situations with a height less than 4 mm using the transcrestal technique, obtaining a 91.9% implant survival rate. However, the transcrestal technique suffers a significant risk of sinus membrane perforation. Nevertheless, studies using the transcrestal technique have shown successful long-term results, while it remains to be seen whether short implants will obtain comparable results over time. Several comparative systematic reviews of short implants in combination with vertical ridge augmentation have not found significant differences between short implants and longer implants placed in areas in which vertical bone regeneration procedures were performed, although differences in the number of surgical complications were reported between the two groups, whereby short implants suffered fewer complications. However, it should be noted that most of these studies involved vertical augmentation in the posterior mandible rather than the posterior maxilla, or were case reports, so further research is necessary in order to clarify and compare results between the two treatment options [38,39,40]. In one of the few randomized clinical trials in the literature, Felice et al. concluded that the use of 5-mm extra-short implants compared with implants of greater length placed at sites where a bone height regeneration procedure had been performed, produced similar outcomes, whether in the maxilla or mandible [41]. 

In addition, extra-short implants showed shorter rehabilitation times, lower economic cost, and fewer complications compared with longer implants [41]. 

All the studies that have compared the use of short implants with transcrestal or side window vertical augmentation techniques have investigated cases of implants replacing single teeth. In the present protocol, which rehabilitated partially edentulous patients, an extra-short implant was splinted to an implant of greater length, so a direct comparison of results with other studies cannot be made. The splinting protocol overcomes the limitation that 4-mm extra-short implants cannot be indicated for rehabilitating single teeth. Splinting implants also reduces biomechanical risks, such as those deriving from the crown-to-implant ratio, which may be unfavorable in some situations. However, most studies investigating crown-to-implant ratios with short and extra-short implants compared with implants longer than 8 mm have found that this factor has no influence on crestal bone stability or implant survival [19,20,21,42,43,44]. It should also be noted that studies using finite element analysis and clinical investigations of crown-to-implant ratio have reported a higher risk of potential prosthetic complications, such as the loosening of structures and possible fractures due to structural fatigue [45,46]. Due to the present study’s limited follow-up, it is not possible to draw conclusions regarding biomechanical complications, although it should be noted that none occurred during the 24-month follow-up period. In an analysis of splinted implants, which presents some similarity to the present protocol, Malmstrom et al. used a splinted implant with implants of up to 6 mm in length splinted to other implants, obtaining similar results to 11-mm implants in the posterior maxilla [47]. Anitua et al. investigated 6.5 mm long implants of different diameters splinted to longer implants with immediate loading in the posterior regions of the maxilla and the mandible and a follow-up period of 14 ± 5 months. No implant losses occurred and the short implants obtained a mean insertion torque of 46 ± 9 Ncm [48]. Another alternative to the present protocol of an extra-short implant splinted to a longer implant could be the incorporation of a cantilever. A review by Zurdo et al. noted that when cantilever-fixed implants fail, the most common reason is implant fracture. Although this only occurred in a low percentage of cases, it is an essential consideration for restoration survival [49].

A recent review by Storelli et al. found that the use of a cantilever can be a successful treatment option in partially edentulous patients, with a complication rate of 26.6% over observation periods of 5–10 years; however, treatments using single-tooth implants with cantilevers have not been sufficiently documented to produce any clear conclusion [50]. Providing the clinical situation permits, treatments using a splinted extra-short implant instead of a cantilever it could offer a viable alternative, as the present work observed no short-term prosthetic or biological complications. Nevertheless, a review by Ravidà et al. found that prosthetic complications observed in case reports appeared after three years or longer, so longer-term results are needed before an adequate comparison can be made [51].

It should also be mentioned that in vitro studies have shown that splinted implants provide better distribution of high occlusal forces despite the unfavorable crown-to-implant ratio [52]. A systematic review by Meijer et al. showed that with splinted implant-supported restorations, the connection between crown and implant may behave differently, perhaps transmitting forces onto peri-implant bone of a different magnitude from non-splinted restorations [53]. In the present study, crestal bone stability was measured by radiographic examination at the time of implant placement, and 2, 6, 12, and 24 months after. The evolution of crestal bone remodeling around the extra short and longer 8-mm implants was assessed from the time of placement to evaluation times at 2, 6, 12, and 24 months. The implant design has a 1.8-mm-long cervical section of machined titanium; in many clinical situations, in order to optimize implant fixation, a part of the machined portion is placed below the bone crest, which may induce different bone remodeling in these specific situations.

The provisionalization phase commenced two months after implant placement, placing temporary abutments and screw-retained acrylic crowns. The final restoration phase was completed six months after implant placement, using Variobase abutments with splinted metal-ceramic crowns screwed and cemented to the Variobase abutments. At the time of publication, no implants have been lost. In a recent report of 6-mm single-tooth implants, Rossi et al. have demonstrated that the use of implants for single-tooth restoration is safe, obtaining marginal bone loss of 0.2 ± 0.4 mm over 5 and 10-year follow-up periods [54]. A systematic review by Tolentino da Rosa de Souza et al. reported marginal bone loss values of 0.1–0.54 mm, but these measurements were taken at the end of shorter follow-up periods [55]. The crestal bone stability results obtained in the present study (0.24 ± 0.23 mm in the 8mm group and 0.33 ± 0.65 mm in the 4mm group) show that remodeling in the two implant groups (8 mm and 4 mm) was similar over time and tended to converge at approximately −0.30 mm. These changes are similar to those reported in other studies, although they need confirmation by longer-term studies using splinted implants. In the posterior maxilla, survival rates for short implants are lower than in the mandible. One reason for this may be the bone characteristics and therefore the implant fixation achieved [56]. In the present study, the surgeons observed (subjectively) that in cases in which the residual bone height at the maxillary sinus floor was 5 mm or higher, the fixation of extra-short implants was more easily achieved; this was recorded in the individual patients’ medical notes.

## 6. Conclusions

Using an 8-mm long implant placed in a mesial position splinted to a 4-mm long implant placed in a distal position obtained promising results with no implant failures during the 24-month follow-up. Despite the short follow-up time and small number of cases treated, the results provide further clinical evidence in favor of the use of extra-short implants. This alternative clinical approach to rehabilitating atrophic posterior maxillae with limited ridge height due to the proximity to the maxillary sinus obtained adequate and promising results, suggesting that this protocol may prove a valid alternative to the currently-established sinus lifting procedures with or without grafting.

## Figures and Tables

**Table 1:** Follow-up visits were planned to take place at two months (provisionalization), 6 months (definitive restoration), 12, and 24 months after implant placement.

Surgery	Suture Removal	Provisionalization	Definitive Restorations	Follow-up
Day 0	Day 15	Two months	Six months	12 and 24 months

**Table 2:** Statistical power according to parameter and evaluation stage.

Parameter Evaluated	Day 0	2 Months	6 Months	12 Months	24 Months
ISQ	**94%**	**94%**	**88%**	**84%**	**60%**
Bone remodeling	**-**	**-**	**-**	**67%**	**53%**

**Table 3:** Mean ISQ stability measurements by time and implant type (L/S).

Implant	Stability (ISQ). Mean ± Standard Deviation				
	Baseline	Two Months	Six Months	12 Months	24 Months
8 mm	64.4 ± 4.9	72.7 ± 1.9	73.8 ± 3.9	78.8 ± 1.4	79.5 ± 1.3
4 mm	54.9 ± 4.9	66.2 ± 5.8	68.1 ± 6.9	74.4 ± 2.9	77.0 ± 2.6
*p-*value (Mann-Whitney)	0.001 *	0.016 *	0.105	0.007 *	0.400

* Statistical differences *p* < 0.05.

**Table 4:** Mean MBL (marginal bone loss) values (mm) ± standard deviation by implant type at each evaluation time.

Implant	MBL (mm). Mean ± Standard Deviation		
	Baseline	12 Months	24 Months
8 mm	1.04 ± 0.78	1.27 ± 0.85	0.87 ± 0.41
4 mm	0.89 ± 0.88	1.37 ± 1.01	1.14 ± 0.95
*p-*Value (Mann-Whitney)	0.739	0.980	1.000

**Table 5:** MBL change (mm) ± standard deviation by stage and implant type.

Implant	MBL Change. Mean ± Standard Deviation (mm)	
	12 Months	24 Months
8-mm	−0.13 ± 0.30	−0.24 ± 0.23
4-mm	−0.39 ± 0.60	−0.33 ± 0.65
*p-*Value (Mann–Whitney)	0.442	1.000
